# Elevated NTCP expression by an iPSC-derived human hepatocyte maintenance medium enhances HBV infection in NTCP-reconstituted HepG2 cells

**DOI:** 10.1186/s13578-021-00641-1

**Published:** 2021-07-05

**Authors:** Xinlei Li, Zhaohui Xu, Bidisha Mitra, Minghang Wang, Haitao Guo, Zongdi Feng

**Affiliations:** 1 Center for Vaccines and Immunity, The Abigail Wexner Research Institute at Nationwide Children’s Hospital, Columbus, OH 43205 USA; 2 Department of Microbiology and Molecular Genetics and UPMC Hillman Cancer Center, University of Pittsburgh, Pittsburgh, PA USA; 3 Department of Pediatrics, The Ohio State University College of Medicine, Columbus, OH USA

**Keywords:** Hepatitis B virus, NTCP, Entry, CMV promoter

## Abstract

**Background:**

The sodium taurocholate cotransporting polypeptide (NTCP) is a functional receptor for hepatitis B virus (HBV). NTCP-reconstituted human hepatoma cells support HBV infection, but the infection is suboptimal and no apparent HBV spread has been observed in this system.

**Results:**

We found that NTCP-reconstituted HepG2 cells were highly susceptible to HBV infection after cells were cultured in a commercial human inducible pluripotent stem cell (iPSC)-derived hepatocyte maintenance medium (HMM). The enhanced HBV infection coincided with increased NTCP expression, and was observed in six different clones of HepG2-NTCP cells. Promoter assays indicated that HMM activated the cytomegalovirus immediate-early (IE) promoter that drives the NTCP expression in the HepG2-NTCP cells. RNA-Seq analysis revealed that HMM upregulated multiple metabolic pathways. Despite highly upregulated NTCP expression by HMM, no obvious HBV spread was observed even in the presence of PEG 8000.

**Conclusions:**

Our data suggest that this particular medium could be used to enhance HBV infection in NTCP-reconstituted hepatocytes in vitro.

**Supplementary Information:**

The online version contains supplementary material available at 10.1186/s13578-021-00641-1.

## Introduction

Hepatitis B virus (HBV) infection leads to a wide spectrum of liver diseases ranging from chronic hepatitis, cirrhosis, and ultimately hepatocellular carcinoma over several decades [1]. The chronic hepatitis B prevalence was estimated around 3.5% worldwide in 2016, and a total of 257 million people are chronically infected with HBV [2]. HBV vaccines have been routinely used in infants and adults and can effectively prevent new infections [3]. However, the current clinically approved HBV antivirals including nucleos(t)ide analogs and interferons are not able to achieve an HBV eradication or functional cure [4–6].

HBV is an enveloped virus and a member of Hepadnaviridae family. It contains a partially double-stranded relaxed circular DNA (rcDNA) genome of approximate 3200 bp in length [7]. After entry into hepatocytes, HBV rcDNA is transported to the nucleus and converted to a covalently closed circular DNA (cccDNA) [8, 9]. The cccDNA is the template for transcription of all viral RNAs including the pregenomic RNA (pgRNA) and four additional mRNA, which are translated into a total of seven viral proteins: the large, middle, and small envelope proteins that form the surface antigen (HBsAg), the core antigen (HBcAg), the e antigen (HBeAg), the HBV polymerase, and the regulatory protein X (HBx) [10, 11]. The pgRNA interacts with the viral polymerase to initiate its encapsidation into the core particles, which are then assembled with envelope proteins and released from cells as virions [12, 13].

A lack of robust cell culture systems and suitable small animal models has hampered the development of novel HBV antivirals or therapeutics [14]. Primary human or Tupaia hepatocytes, HepaRG, and hepatoma cell lines stably transfected with an overlength HBV genome (HepG2.2.15, HepAD38, or HepDE19) have been widely used for in vitro HBV basic and antiviral research [15–24]. However, these systems have limitations including the high batch-to-batch variability of primary hepatocytes, long-term differentiation procedure for HepaRG cells, and the incomplete viral life cycle in HBV stably transfected cell lines. Embryo stem cell or induced pluripotent stem cell (iPSC)-derived hepatocyte-like cells are also susceptible to HBV infection, but the process of generating these cells is time-consuming and labor-intensive [25–27]. In 2012, sodium taurocholate cotransporting polypeptide (NTCP), also known as SLC10A1, was identified to be a cell receptor for HBV and its satellite virus, hepatitis D virus (HDV) [28–30]. Since then, several NTCP-reconstituted human or murine hepatic cell lines have been generated and used for studying HBV infection [31, 32]. Particularly, the HepG2-NTCP cells have been the most widely used cells by HBV researchers. However, NTCP expression cannot maintain at a high level in proliferative cultured hepatocytes, or in livers undergo regeneration [33, 34]. It may involve the elevated NTCP degradation through the ubiquitin–proteasome system and/or lysosome, or the decreased transcription mediated by some important transcriptional factors like p53 [34].

In the present study, we reported that a commercial hepatocyte maintenance medium (HMM) that significantly enhances HBV infection efficiency by only treating the HepG2-NTCP cells for 24 h prior to virus inoculation. The enhancement of HBV infection was correlated with elevated NTCP expression.

## Materials and methods

### Virus and cells

HepG2-NTCP cells were previously established and cultured with Dulbecco’s modified Eagle’s medium (DMEM) supplemented with 10% fetal bovine serum (FBS), 1% penicillin and streptomycin (D10), and blasticidin (8 μg/ml) [35]. HepG2 cells were maintained in the same medium aforementioned without blasticidin. HBV particles were collected from HepDE19 cell culture supernatants as described previously [35, 36]. Briefly, HepDE19 cells were cultured in Dulbecco’s modified Eagle’s medium (DMEM) supplemented with 10% tetracycline-free fetal bovine serum (FBS) and 1% penicillin and streptomycin to induce HBV virion production, supernatants were collected every other day for 18 days. The pooled supernatants were mixed with polyethylene glycol (PEG)-8000 (final concentration of 10%) and rotated at 4 °C for overnight, followed by centrifugation at 1000×*g* for 30 min at 4 °C. The viral particles were resuspended in DMEM medium, the stocks were aliquoted, quantified, and stored at − 80 °C.

### Reagents

Hepatocyte maintenance medium (HMM) (Y30051, TaKaRa Bio USA) was used to pretreat or for long-term culture of HepG2 and HepG2-NTCP cells. Primary hepatocyte maintenance medium (PMM) was prepared according to previous publications [27, 35]. The NTCP antibody was generously given by Dr. Bruno Stieger (Department of Chemistry and Applied Biosciences, University of Zurich, Switzerland). HBc (B058601-1, Agilent) and HBs (MD-05-0186, RayBiotech) antibodies were used to stain HBV viral proteins for immunofluorescence assay. Cyclosporine A (30024, MilliporeSigma), a chemical that reportedly targets NTCP to inhibit HBV infection was used.

### HBV infection

HepG2 or HepG2-NTCP cells were pretreated with HMM for 24 h before inoculating with HBV at different doses mixed with DMEM containing 4% PEG8000 for overnight. The inoculum was removed and HMM or D10/2%DMSO medium was used for further cell culture.

### Immunofluorescence assay

At different days post-HBV infection, the cells were fixed with 4% paraformaldehyde at room temperature for 20 min. The fixed cells were blocked, permeabilized, stained with specific primary and then secondary antibodies, counterstained with DAPI, and photographed with an LSM 800 microscope (Carl Zeiss Inc., Thornwood, NY, United States).

### RNA-Seq analysis

HepG2-NTCP cells were cultured with D10/2%DMSO or HMM for 24 h, followed by total RNA extraction with RNeasy Isolation kit (Qiagen) and treated with DNase I. Following assessment of the quality of total RNA using an Agilent 2100 Bioanalyzer and RNA Nano chip (Agilent Technologies), 150 ng total RNA was treated to deplete the levels of ribosomal RNA (rRNA) using target-specific oligos combined with rRNA removal beads. Following rRNA removal, mRNA was fragmented and converted into double stranded cDNA. Adaptor-ligated cDNA was amplified by limit cycle PCR. After library quality was determined via Agilent 4200 TapeStation and quantified by KAPA qPCR, approximately 60 million paired-end 150 bp sequence reads were generated on the Illumina HiSeq 4000 platform. For data analysis, each sample was aligned to the GRCh38.p13 assembly of the human reference from GENCODE using version 2.1.0 of the RNA-Seq aligner HISAT2. Features were identified from the GTF file from GENCODE. Feature coverage counts were calculated using fetureCounts and differentially expressed features were calculated using DESeq2 package in R and significantly differential expressions were those with a false discovery rate (FDR) of  <  0.05 and fold-change of  >  2.

### Gene ontology and pathway enrichment analyses

Grouping of differentially expressed gene (DEG) dataset into molecular functions, biological processes, or cellular components was achieved by gene ontology (GO) analysis based on the DAVID program. The Kyoto Encyclopedia of Genes and Genomes (KEGG) was used to understand high-level biological functions and utilities.

## Results

### HMM enhances HBV infection at the entry step

**Fig. 1:**
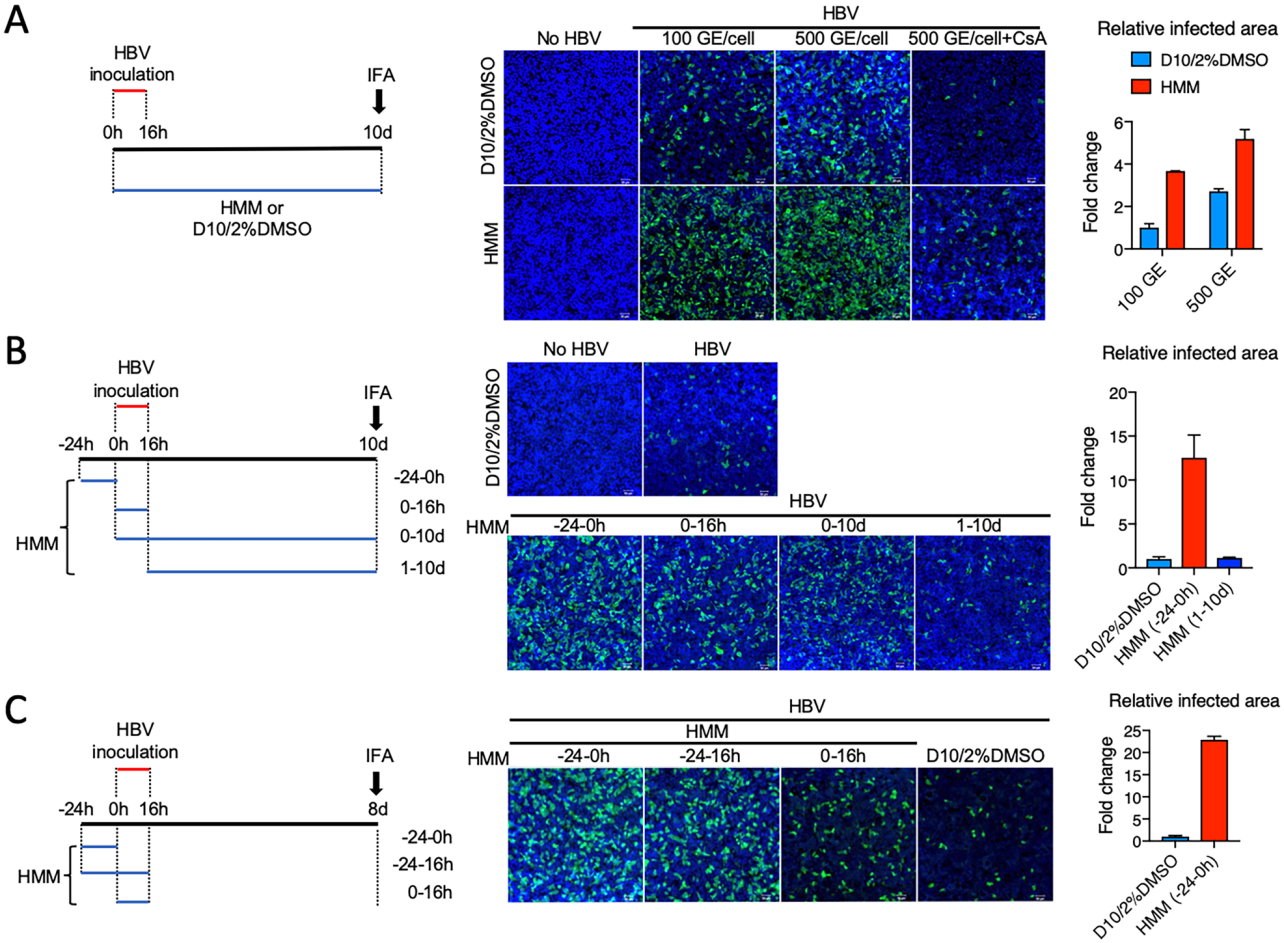
Hepatocyte maintenance medium (HMM) enhances HBV infection in HepG2-NTCP cells. **A** HepG2-NTCP cells were inoculated with HBV at 100 or 500 genome equivalents (GE)/cell diluted in 4% PEG8000-containing DMEM or HMM in the presence or absence of 10 μM cyclosporin A (CsA). After 16 hours (h), the inoculum was removed, and cells were washed and refed with either DMEM supplemented with 10%FBS and 2%DMSO (D10/2%DMSO) or HMM. At 10 days (d) post-inoculation, cells were fixed and subjected to immunofluorescence assays using an anti-HBc antibody. Nuclei were stained with DAPI. **B**, **C** HMM was added to the HepG2-NTCP cell culture before, during, or after HBV inoculation (100 GE/cell). At 10 d (**B**) or 8 d (**C**) post-inoculation, cells were fixed and stained with an anti-HBc antibody. Scale bar  =  50 μm. HBV-infected areas were quantified using the ImageJ software (NIH), and data were normalized to values in culture infected with 100 GE/cell of HBV in the regular medium (D10/2% DMSO) and presented as mean  ±  SEM from two independent experiments

In a process of evaluating the utility of induced pluripotent cell (iPSC)-derived hepatocyte-like cells (HLCs) in studying HBV infection, we serendipitously found that a commercial hepatocyte maintenance medium (HMM) designed to maintain iPSC-HLCs appeared to enhance HBV infection of HepG2-NTCP cells when compared to the regular medium D10/2%DMSO (DMEM supplemented with 10% FBS and 2% DMSO; Fig. 1A). HBV Infection of these cells strictly depended on the transgenic NTCP, as adding during virus inoculation with cyclosporin A, a known HBV entry inhibitor targeting NTCP [37–39], resulted in a great reduction in the number of infected cells. To determine which stage(s) of HBV infection was enhanced by HMM, HepG2-NTCP cells were cultured in HMM either before, during, or after HBV inoculation. Culturing the HepG2-NTCP cells in HMM for 24 h before HBV inoculation yielded the greatest enhancement of infection. In contrast, culturing the cells in HMM after HBV inoculation did not enhance the infection (Fig. 1B). Interestingly, culturing the cells in HMM before and during HBV inoculation did not lead to further enhancement (Fig. 1C). Quantification of HBV infected area by ImageJ revealed up to 23-fold increase when HepG2-NTCP cells were first cultured in HMM for 24 h before HBV inoculation (relative to cells cultured continuously in regular medium). These results demonstrate that the use of HMM greatly improved HBV infection in HepG2-NTCP cells. Since the greatest effect was observed when HMM was used prior to HBV inoculation, HMM likely enhanced HBV infection at the entry level.

### HMM promotes HBV infection in HepG2-NTCP cells by increasing NTCP expression

**Fig. 2:**
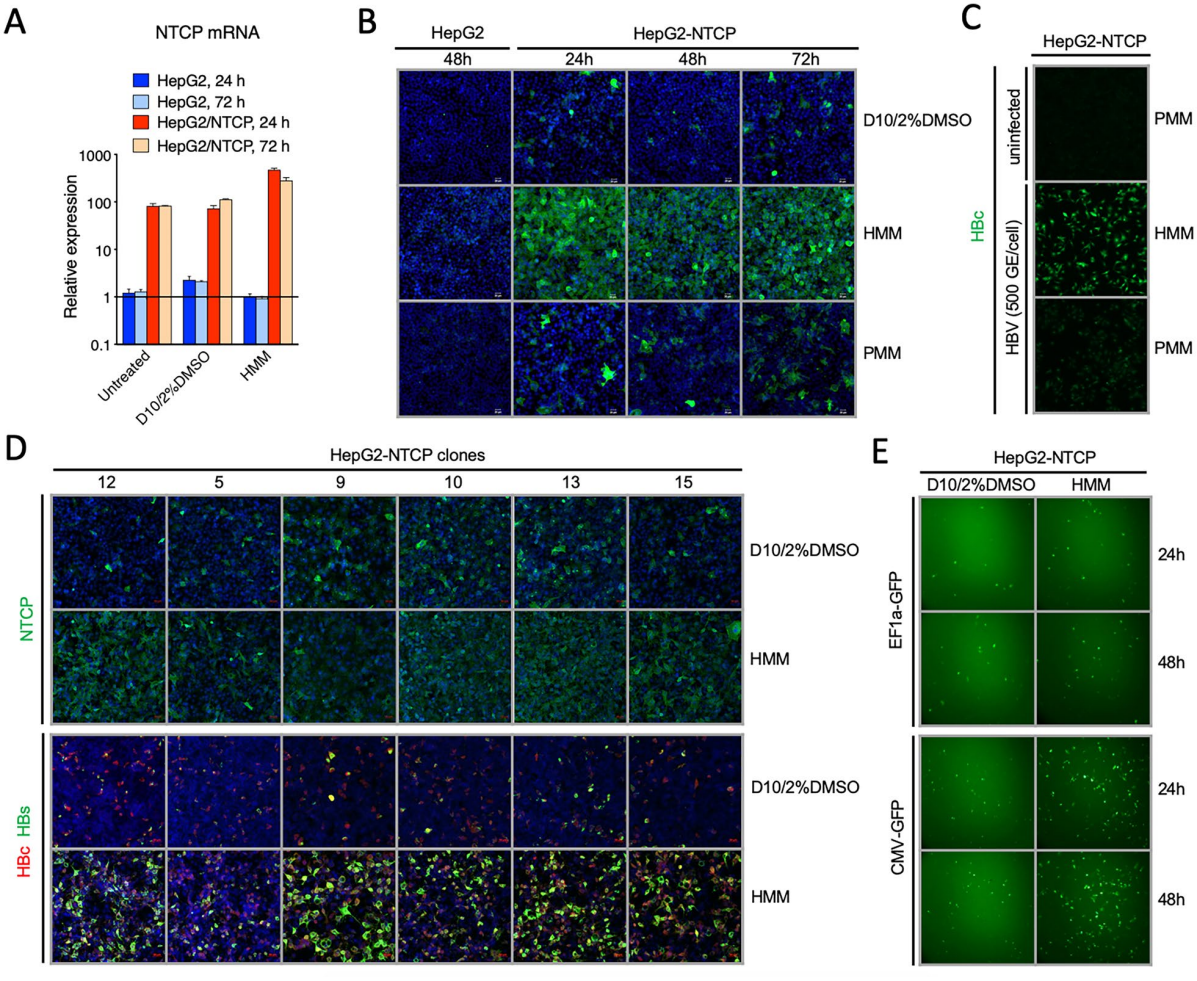
Ectopic NTCP is induced by HMM treatment. **A** RT-qPCR of NTCP mRNA levels in HepG2 and HepG2-NTCP cells after being culturing with D10/2%DMSO medium or HMM for 24 or 72 h. Untreated, D10 medium without 2% DMSO. **B** Immunofluorescence images of NTCP protein expression in HepG2-NTCP or parental HepG2 cells after being cultured in indicated medium for 24, 48, or 72 h. Primary hepatocyte maintenance medium (PMM) contains 10% FBS, 1% Penicillin/Streptomycin, 0.17 μM of human Insulin, 10 μM of hydrocortisone 21-hemisuccinate and 1.8% DMSO [31]. **C** HepG2-NTCP cells were left uninfected, or pretreated with HMM or PMM for 16 h before inoculating with HBV (500 GE/cell) in PMM containing 4% PEG 8000 for 24 h. The HBV inocula were removed and cells were maintained in PMM for 7 days. **D** Expression of NTCP, HBc (red) and HBs (green) in different HepG2-NTCP clones at 10 d post-HBV infection. Clone 12 was used in most experiments in this study. **E** GFP expression in HepG2-NTCP cells transfected with GFP-encoding plasmids under the control of either a CMV-IE promoter or an EF1α promoter and subsequently cultured in indicated medium for indicated times. Scale bar  =  50 μm

Next, we examined if HMM caused a change in NTCP expression in HepG2-NTCP cells. The NTCP mRNA level was significantly increased at both 24 and 72 h after cells were cultured in HMM (Fig. 2A). Similarly, the NTCP protein level was also increased at 24, 48, or 72 h after switching the regular medium to HMM (Fig. 2B). However, the endogenous NTCP was not induced in the parental HepG2 cells under the same condition. Since several studies have used primary hepatocyte maintenance medium (PMM) for culturing NTCP-reconstituted hepatoma cells during HBV infection [30, 35, 40, 41], we also examined if PMM that was previously used by another group to maintain hepatocytes derived from human stem cells [27] had any enhancement effect on NTCP expression similar to HMM. No further induction of NTCP was observed when HepG2-NTCP cells were cultured in PMM, indicating that the effect of HMM on NTCP expression was unique. In addition, HBV also exhibited higher infectivity in HepG2-NTCP cells pretreated by HMM compared to PMM (Fig. 2C). Since the HepG2-NTCP cells were originally generated by single cell cloning, five additional HepG2-NTCP stable cell clones were tested. NTCP expression and HBV infection were enhanced by HMM in all cell clones tested (Fig. 2D). Thus, the effect of HMM on NTCP expression was not cell clone-specific.

Because the reconstituted NTCP in HepG2-NTCP cells is driven by a CMV-immediate early (IE) promoter, we next examined if HMM enhances CMV-IE promoter activity using a reporter assay. Plasmids containing a GFP cassette controlled by either a CMV-IE or an EF1α promoter were transfected into HepG2-NTCP cells, and the expression of GFP was examined under a fluorescence microscope after culturing cells in HMM. Only CMV-IE promoter-controlled GFP expression was enhanced by HMM (Fig. 2E), suggesting that HMM increases CMV-IE promoter activity.

### HMM induced transcriptomic changes in HepG2-NTCP cells

**Fig. 3:**
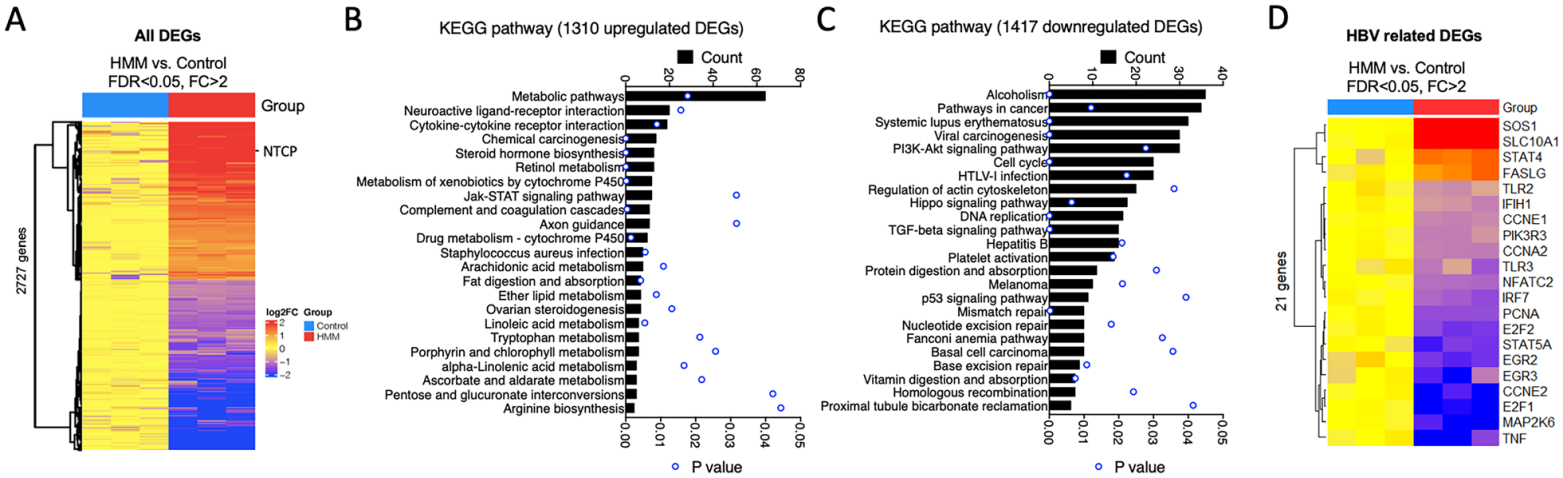
Transcriptome analysis of HMM-treated HepG2-NTCP cells. HepG2-NTCP cells were treated with HMM or D10/2%DMSO medium (control) for 24 h. Total RNA was collected for RNA-Seq analysis. **A** Heatmap of 2727 differentially expressed genes (DEGs, foldchange  >  2, adjusted p value  <  0.05) identified in HMM versus control group. KEGG pathway analyses of 1310 upregulated (**B**) and 1417 downregulated (**C**) DEGs. **D** Heatmap of differentially expressed HBV-related genes (KEGG ID hsa05161) in HMM versus control group

To investigate whether the enhanced NTCP expression was solely responsible for HMM-mediated enhancement of HBV infection in HepG2-NTCP cells, we performed RNA-Seq analysis to profile the transcriptomic changes caused by HMM. A total of 2,727 differentially expressed genes (DEGs) were identified, including 1310 upregulated and 1417 downregulated DEGs (Fig. 3A; Additional file 1: Table S1). Consistent with the qRT-PCR result in Fig. 2A, the NTCP reads were increased by 6.4-fold after cells were cultured in HMM for 24 h (Fig. 3A). KEGG pathway analysis revealed metabolic pathways, neuroactive ligand-receptor interaction, and cytokine-cytokine receptor interaction as the top three pathways activated by HMM (Fig. 3B). HMM also enhanced metabolic activities in the HepG2-NTCP cells, as evidenced by the enriched KEGG pathways associated with mature hepatocyte functions including lipid metabolism, amino acid synthesis, and xenobiotics metabolism (Fig. 3B). By contrast, alcoholism, pathways in cancer, and systemic lupus erythematosus were the most enriched pathways for the 1417 downregulated DEGs (Fig. 3C). Among the 162 HBV-related genes (KEGG pathway hsa05161), the upregulated genes included SOS1, SLC10A1 (NTCP), STAT4, and FASLG; while 17 genes were downregulated, among which TNF, MAP2K6, E2F1, and CCNE2 were the most downregulated ones (Fig. 3D). Of note, TNF is capable of inhibiting HBV replication through disruption of capsid Integrity [42], and downregulation of E2F1 or CCNE2 could lead to cell cycle arrest, which may be beneficial for HBV replication [43]. However, since the maximal effect of HMM on HBcAg and HBsAg expression was achieved by HMM addition prior to HBV inoculation, increasing HBV entry via NTCP upregulation appears to be the primary mechanism by which HMM enhances HBV infection.

### Long-term culture in HMM does not lead to HBV spread

**Fig. 4:**
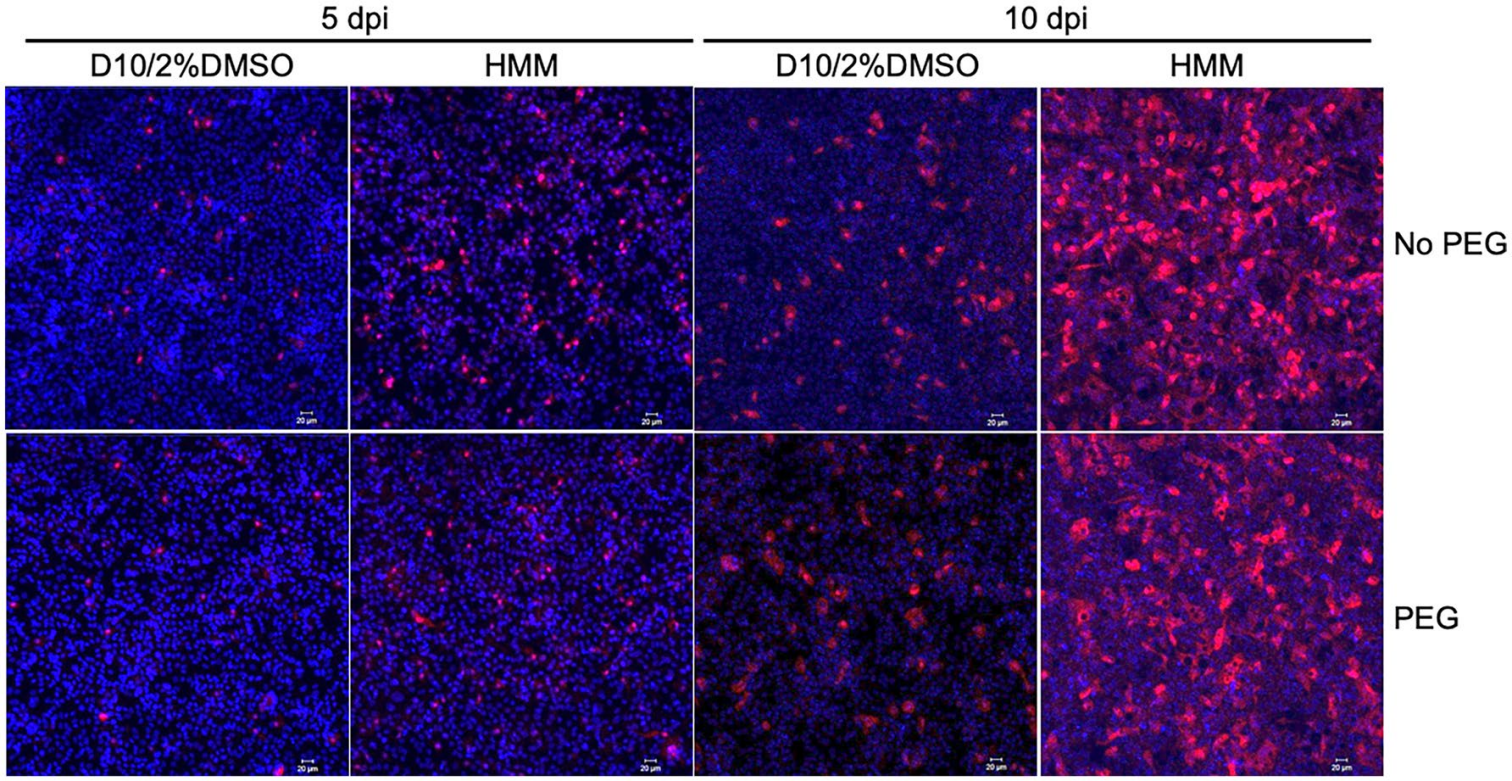
Combination of HMM with PEG does not lead to HBV spread in HepG2-NTCP cells. HepG2-NTCP cells were pretreated with HMM or D10/2%DMSO for 24 h prior to HBV infection. After removal of the inoculum, cells were subsequently cultured in HMM or D10/2%DMSO medium alone or supplemented with 4% PEG8000 for 5 or 10 days. The HBV infection rate was measured by immunofluorescence with HBc antibody. Scale bar  =  50 μm

Despite a substantial and sustained increase in NTCP expression induced by HMM, addition of HMM after HBV inoculation did not significantly increase the number of infected cells at 10 days post-inoculation when compared to cells cultured in the regular medium (Fig. 1B). This indicated that increasing NTCP expression alone is not sufficient for HBV spread in HepG2-NTCP cells. Since polyethylene glycol (PEG) has been routinely used to increase HBV attachment to cells and one report shows that the continuous presence of PEG could result in HBV spread in cell cultures [44], we tested if combination of PEG and HMM facilitates HBV spread. HepG2-NTCP cells were cultured in HMM or D10/2%DMSO for 24 h, followed by HBV inoculation for 16 h in the presence of 4% PEG8000. After the removal of the inoculum, cells were cultured with either HMM or D10/2%DMSO in the presence or absence of 4% PEG8000. Although more cells were infected when cultured in HMM, addition of PEG8000 did not lead to further increase in the number of HBV infected cells (Fig. 4).

## Discussion

Despite the wide use of NTCP-reconstituted hepatoma cells for HBV research, the low infection efficiency remains a major hurdle. In this study, we describe an effective way to enhance HBV infection in NTCP-reconstituted HepG2 cells. A brief (24 h) treatment with a commercially available hepatocyte maintenance medium (HMM) prior to HBV inoculation resulted in a substantial increase in HBV infection. This enhancement caused by HMM was due to an unexpected boosting effect on the CMV IE promoter which drives NTCP expression in these cells. Given that increased NTCP expression and HBV infection was observed in multiple independent cell clones, and that consistent results were obtained with different batches of HMM, this method is likely generally applicable to other cell culture systems reconstituted with CMV IE promoter-driven NTCP to enhance HBV infection.

The expression of NTCP in hepatocytes is critically dependent on their differentiation and proliferation status [45]. Primary hepatocytes express high levels of NTCP, but rapidly lose NTCP expression upon isolation and culture in vitro [30]. Commonly used hepatoma cell lines including HepG2, Huh7, and HepaRG cells have barely detectable NTCP under normal proliferative conditions. Nonetheless, upon DMSO-induced differentiation, HepaRG cells regain NTCP expression and become susceptible to HBV infection [24]. Interestingly, cell cycle arrest promotes NTCP expression and localization to cell membrane in NTCP-reconstituted HepG2 cells, thereby increasing the susceptibility to HBV infection [34]. The HMM used in this study was originally designed to maintain the hepatocyte phenotype of iPSC-derived hepatocyte-like cells. HMM also contains DMSO, consistent with the cytostatic effect we observed when HepG2-NTCP cells were culture in HMM for longer periods. However, DMSO alone was apparently insufficient to increase NTCP expression in HepG2-NTCP cells. The PMM used by others also did not increase NTCP expression in these cells, highlighting the uniqueness of HMM in enhancing NTCP expression and HBV infection.

The induction of NTCP expression in HepG2-NTCP cells by HMM was rather quick (< 24 h), and prolonged culture in HMM did not further increase NTCP expression. Our RNA-Seq analysis indicated that multiple metabolic pathways are activated by HMM. However, how exactly HMM enhances CMV IE promoter activity remains unknown. The CMV IE promoter and enhancer contains binding sites for multiple transcription factors including NFκB, SP1, ETS, C/EBP, SRF, YY1, NF1, Gfi1, SBP [46], and the expression of ETS2, CEBPB, NFKB2, SP1, and NF1 were induced in HMM-treated cells. Identifying transcription factors activated by HMM will offer a better understanding of how HMM activates CMV promoter and modulates cellular pathways resulting in enhanced HBV infection.

The active components in HMM that enhanced NTCP expression and HBV infection remain to be identified. HepG2-NTCP cells require pretreatment with optimal medium to gain high susceptibility to HBV infection [35, 47]. DMSO is a common component in these medium, and other molecules including transferrin, EGF, insulin, hydrocortisone, dexamethasone, and selenite may also be beneficial [30, 35]. A recent study by Xiang et al. [45] describes that a combination of five chemicals (5C) can effectively maintain the mature function of cultured primary human hepatocytes (including high levels of NTCP expression) up to 30 days. Although the composition of HMM is not revealed to us, this information is not necessary for using HMM to enhance HBV infection.

In summary, we reported that a commercially available hepatocyte maintenance medium (HMM) can significantly improve HBV infection in NTCP-reconstituted HepG2 cells. This provides an easy and effective way to establish robust HBV infection, which will aid in the basic and antiviral research of HBV.

## Supplementary Information

**Additional file 1: Table S1.** Differentially expressed genes in HepG2-NTCP cells following HMM treatment.

## Data Availability

The RNA-Seq data has been uploaded to the GEO database (Accession No. GSE173576).
